# Effects of long-term aspirin use on molecular alterations in precancerous gastric mucosa in patients with and without gastric cancer

**DOI:** 10.1038/s41598-017-13842-x

**Published:** 2017-10-17

**Authors:** Yuki Michigami, Jiro Watari, Chiyomi Ito, Ken Hara, Takahisa Yamasaki, Takashi Kondo, Tomoaki Kono, Katsuyuki Tozawa, Toshihiko Tomita, Tadayuki Oshima, Hirokazu Fukui, Takeshi Morimoto, Kiron M. Das, Hiroto Miwa

**Affiliations:** 1 Division of Gastroenterology, Department of Internal Medicine, Hyogo College of Medicine, Nishinomiya, Japan; 2 Department of Clinical Epidemiology, Hyogo College of Medicine, Nishinomiya, Japan; 3 Division of Gastroenterology and Hepatology, Departments of Medicine and Pathology, Robert Wood Johnson Medical School, Rutgers, Cancer Institute of New Jersey, New Brunswick, United States

## Abstract

The risk of gastric cancer (GC) remains even after *H. pylori* eradication; thus, other combination treatments, such as chemopreventive drugs, are needed. We evaluated the effects of aspirin on genetic/epigenetic alterations in precancerous conditions, i.e., atrophic mucosa (AM) and intestinal metaplasia (IM), in patients with chronic gastritis who had taken aspirin for more than 3 years. A total of 221 biopsy specimens from 74 patients, including atrophic gastritis (AG) cases without aspirin use (control), AG cases with aspirin use (AG group), and GC cases with aspirin use (GC group), were analyzed. Aspirin use was associated with a significant reduction of *CDH1* methylation in AM (OR: 0.15, 95% CI: 0.06–0.41, p = 0.0002), but was less effective in reversing the methylation that occurred in IM. Frequent hypermethylation including that of *CDH1* in AM increased in the GC group compared to the AG group, and *CDH1* methylation was an independent predictive marker of GC (OR: 8.50, 95% CI: 2.64–25.33, p = 0.0003). In patients with long-term aspirin use, the changes of molecular events in AM but not IM may be an important factor in the reduction of cancer incidence. In addition, methylation of the *CDH1* gene in AM may be a surrogate of GC.

## Introduction

*Helicobacter pylori* (*H. pylori*) infection causes non-atrophic gastritis, which progresses to atrophic gastritis, intestinal metaplasia (IM), dysplasia, and finally, gastric cancer (GC)^1^. Thus, the International Agency for Research on Cancer has concluded that *H. pylori* is a class I human carcinogen^2^. To date, some meta-analyses have shown that *H. pylori* eradication reduced the risk of GC in patients with chronic gastritis who underwent endoscopic resection (ER) for early GC^3–7^. However, a recent study from Japan showed that even after *H. pylori* infection was cured and gastric inflammation was eliminated, there was still a risk of GC in the long-term^8^. Additionally, metachronous GC occurred to some degree in patients who had *H. pylori* infection eradicated following ER for early GC^9–13^. Thus, it remains controversial if *H. pylori* eradication suppresses the development of GC. To reduce the risk of GC after *H. pylori* eradication, other combination treatments such as anti-inflammatory agents and dietary or nutritional intervention are needed.

Some studies including meta-analyses have reported that aspirin and other nonsteroidal anti-inflammatory drugs (NSAIDs) are associated with a reduced risk of both colorectal cancer and GC^14–17^. Their anti-carcinogenetic effects have been attributed to inhibition of the cyclooxygenase pathway and their anti-inflammatory abilities^18,19^. The roles of a number of genetic and epigenetic alterations, including microsatellite instability (MSI) and promoter hypermethylation of multiple tumor-related genes, are reportedly involved in GC and precancerous conditions of the stomach^20–33^. The CpG island methylator phenotype (CIMP), characterized by extensive hypermethylation of multiple CpG islands within the genome, is currently recognized as one of the major mechanisms in GC^30^. In addition, IM with a colonic phenotype, as detected by the Das-1 monoclonal antibody (mAb), was shown to be strongly associated with GC^32–35^. However, to date, no study has compared changes in molecular phenotype in patients with chronic gastritis with or without GC who have taken aspirin on a long-term basis.

It was recently reported that the risk of GC was reduced in patients who took aspirin on a regular basis for more than 3 years^16^. In this study, we examined the effects of aspirin on genetic and epigenetic alterations, as well as mAb Das-1 reactivity in precancerous conditions, i.e., atrophic mucosa (AM) and IM, in patients with chronic gastritis who regularly took aspirin for more than 3 years. We also determined the molecular markers linked to carcinogenesis risk in those patients. Finally, we compared the differences in molecular abnormalities between patients with AM and IM.

## Results

### Patient characteristics

The characteristics of the patients are shown in Table 1. The mean duration of aspirin use was 6.3 ± 2.7 years (range 3–15 years) in the atrophic gastritis (AG) group and 6.5 ± 3.4 years (3–15 years) in the GC group; thus there was no significant difference between groups. There was also no significant difference in mean age among the three groups, although there were more males in the GC group than in the controls (*p* = 0.002). *H. pylori* infection rate was not significantly different among the three groups; 24 individuals (75.0%) in the control group, 21 patients (87.5%) in the AG group, and 11 patients (61.1%) in the GC group were negative for *H. pylori*. Of these patients, 21 cases in the control group, 6 in the AG group and 3 in the GC group had undergone *H. pylori* treatment. The remaining 26 patients had not been treated for *H. pylori* infection; thus, their infection was considered naturally eradicated. Of these patients, severe mucosal atrophy (open type according to the endoscopic classification by Kimura and Takemoto)^36^ was identified in 66.7% (2 of 3) of cases in the control group, 60.0% (9 of 15) of cases in the AG group and in 75.0% (6 of 8) of cases in the GC group.

**Table 1:** Patient characteristics.

	Control		AG group		GC group		*P*
	n = 32		n = 24		n = 18		
Past period aspirin use ± SD (yr) (range)	—		6.3 ± 2.7 (3–15)		6.5 ± 3.4 (3–15)		0.67
Medication							
Low-dose aspirin	—		20	(83.3)	18	(100)	0.12
NSAIDs	—		4	(16.7)	0	(0)	
Mean age ± SD (yr)	70.9 ± 8.4		73.0 ± 9.1		74.8 ± 5.9		0.25*
Male: Female	14: 18		15: 9		16: 2		^a^0.002
*H. pylori* infection							
Positive	8	(25.0)	3	(12.5)	7	(38.9)	0.14
Negative	24	(75.0)	21	(87.5)	11	(61.1)	
(Post-eradicated)	(21)		(6)		(3)		

AG, atrophic gastritis; GC, gastric cancer; NSAID, nonsteroidal anti-inflammatory drug.

*Kruskal-Wallis test.

^a^ *P* value comparing the control and GC groups.

### MSI and epigenetic alterations

The incidence of MSI and hypermethylation of seven genes are shown in Tables 2, 3, 4 and 5. Most of the molecular alterations, with the exception of E-cadherin (*CDH1*) methylation, were more frequently found in IM compared to AM in each group (i.e., control, AG, and GC groups).

1. Molecular changes in AM by aspirin use
  The methylation of *CDH1* and methylated-in-tumor-31 (MINT31) in AM significantly decreased in the AG group compared to the control group (*p* < 0.0001 and *p* = 0.03, respectively). Multivariate analysis showed that aspirin use was associated with a significant reduction of *CDH1* gene methylation (odds ratio [OR]: 0.15, 95% confidence interval [CI]: 0.06–0.41, *p* = 0.0002) (Table 2). In contrast, the frequency of methylation of *CDH1* gene, and MINT1 and MINT31 loci significantly increased in the GC group (*p* < 0.0001, *p* = 0.02, and *p* = 0.004, respectively). Multivariate logistic regression analysis showed that *CDH1* methylation was an independent risk factor of significant gastric dysplasia (OR: 8.50, 95% CI: 2.64–25.33, *p* = 0.0003) (Table 3). Also, when adjusting for gender in multivariate analysis, a similar result was obtained (OR: 7.71, 95% CI: 2.34–25.42, *p* = 0.0008). The sensitivity, specificity, positive predictive value, and negative predictive value of *CDH1* methylation for the development of gastric dysplasia were 59%, 86%, 68%, and 80%, respectively.
2. Molecular changes in IM by aspirin use
  The frequency of CIMP in IM significantly decreased in the AG group compared to the control group after aspirin use (*p* = 0.02) (Table 4). On the other hand, although CIMP rate tended to be higher in the GC group than in the AG group (*p* = 0.08), no significant differences in other molecular events between the two groups were found (Table 5).
3. Comparison of molecular events in AM and IM in different parts of the stomach in the AG and GC groups

**Table 2:** Molecular changes in the AM - Comparison of molecular events between the control and AG groups.

No. of biopsy specimens	Control		AG group		*P*	Multivariate analysis			
	n = 63	(%)	n = 56	(%)		OR (95% CI)	*P*	Gender-adjusted OR (95% CI)	*P*
MSI	1	(1.6)	4	(7.1)	0.19				
CIMP	4	(6.3)	2	(3.6)	0.68				
*CDH1*	34	(54.0)	8	(14.3)	< 0.0001	0.15 (0.06–0.41)	0.0002	0.12 (0.04–0.36)	0.0001
*CDKN2A*	1	(1.6)	1	(1.8)	1				
*MLH1*	0	(0)	0	(0)	1				
*MGMT*	0	(0)	1	(1.8)	0.47				
MINT1	11	(17.5)	5	(8.9)	0.19				
MINT31	10	(15.9)	2	(3.6)	0.03	0.80 (0.14–4.86)	0.80	0.93 (0.15–5.48)	0.91
*RUNX3*	0	(0)	0	(0)	1				

AM, atrophic mucosa; AG, atrophic gastritis; OR, odds ratio; CI, confidence interval; MSI, microsatellite instability; CIMP, CpG island methylator phenotype.

**Table 3:** Molecular changes in the AM - Comparison of molecular events between the AG and GC groups.

No. of biopsy specimens	AG group		GC group		*P*	Multivariate analysis			
	n = 56	(%)	n = 26	(%)		OR (95% CI)	*P*	Gender-adjusted OR (95% CI)	*P*
MSI	4	(7.1)	2	(9.1)	1				
CIMP	2	(3.6)	3	(11.5)	0.32				
*CDH1*	8	(14.3)	17	(65.4)	<0.0001	8.50 (2.64–25.33)	0.0003	7.71 (2.34–25.42)	0.0008
*CDKN2A*	1	(1.8)	0	(0)	1				
*MLH1*	0	(0)	0	(0)	1				
*MGMT*	1	(1.8)	0	(0)	1				
MINT1	5	(8.9)	8	(30.8)	0.02	1.26 (0.24–6.55)	0.78	1.32 (0.26–6.78)	0.74
MINT31	2	(3.6)	7	(26.9)	0.004	4.08 (0.55–30.32)	0.11	4.30 (0.54–34.36)	0.17
*RUNX3*	0	(0)	0	(0)	1				

AM, atrophic mucosa; AG, atrophic gastritis; GC, gastric cancer; OR, odds ratio; CI, confidence interval; MSI, microsatellite instability; CIMP, CpG island methylator phenotype

**Table 4:** Molecular changes in the IM - Comparison of molecular events between the control and AG groups.

No. of biopsy specimens	Control		AG group		*P*
	n = 33	(%)	n = 16	(%)	
MSI	5	(15.2)	2	(12.5)	1
CIMP	9	(27.3)	0	(0)	0.02
*CDH1*	7	(21.2)	1	(6.3)	0.25
*CDKN2A*	1	(3.0)	0	(0)	1
*MLH1*	0	(0)	0	(0)	1
*MGMT*	0	(0)	1	(6.3)	0.33
MINT1	23	(69.7)	12	(75.0)	1
MINT31	16	(48.5)	8	(50.0)	1
*RUNX3*	12	(36.4)	2	(12.5)	0.10

IM, intestinal metaplasia; AG, atrophic gastritis; MSI, microsatellite instability; CIMP, CpG island methylator phenotype.

**Table 5:** Molecular changes in the IM - Comparison of molecular events between the AG and GC groups.

No. of biopsy specimens	AG group		GC group		*P*
	n = 16	(%)	n = 27	(%)	
MSI	2	(12.5)	5	(18.5)	0.69
CIMP	0	(0)	6	(20.0)	0.08
*CDH1*	1	(6.3)	4	(13.3)	0.64
*CDKN2A*	0	(0)	0	(0)	1
*MLH1*	0	(0)	0	(0)	1
*MGMT*	1	(6.3)	1	(3.3)	1
MINT1	12	(75.0)	19	(63.3)	0.52
MINT31	8	(50.0)	14	(46.7)	1
*RUNX3*	2	(12.5)	10	(33.3)	0.17

IM, intestinal metaplasia; AG, atrophic gastritis; GC, gastric cancer; MSI, microsatellite instability; CIMP, CpG island methylator phenotype.

**Figure 1:**
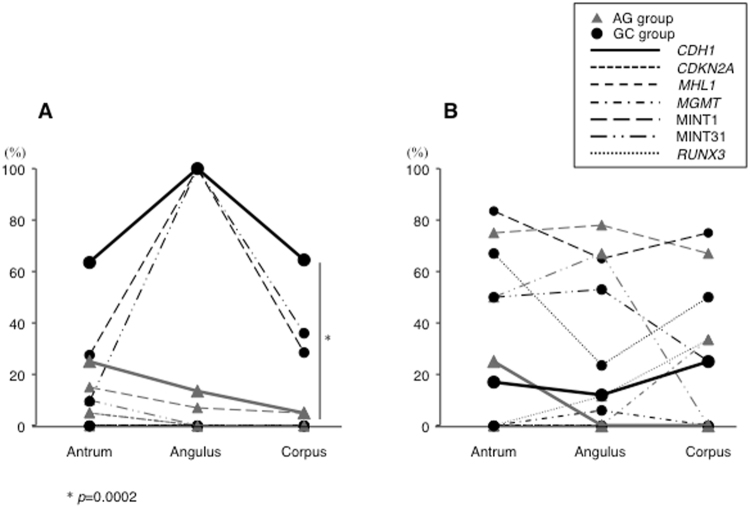
CpG island methylation in precancerous conditions in three different parts of the stomach (antrum, angulus, and corpus). (**A**) In AM, hypermethylation of *CDH1* gene, and MINT1 and MINT31 loci was observed throughout the stomach in the AG and GC groups. *CDH1* methylation in AM, a predictive marker for gastric dysplasia, was significantly higher at the greater curvature of corpus in the GC group than in the AG group (*p* = 0.0002). (**B**) In IM, DNA hypermethylation of most genes other than *CDH1* in the AG group and *CDKN2A* and *MLH1* in the GC group was seen in the various portions of the stomach.

Methylation of *CDH1*, MINT1 and MINT31 in AM was observed in the stomach in both the AG and GC groups. Similarly, in IM, CpG island hypermethylation of most of the genes analyzed was identified in different portions of the stomach in both groups (Fig. 1A and B). The frequency of *CDH1* methylation in AM was significantly higher in biopsy specimens taken from the greater curvature of the corpus in the GC group than in those from the AG group (*p* = 0.0002).

### mAb Das-1 reactivity

**Figure 2:**
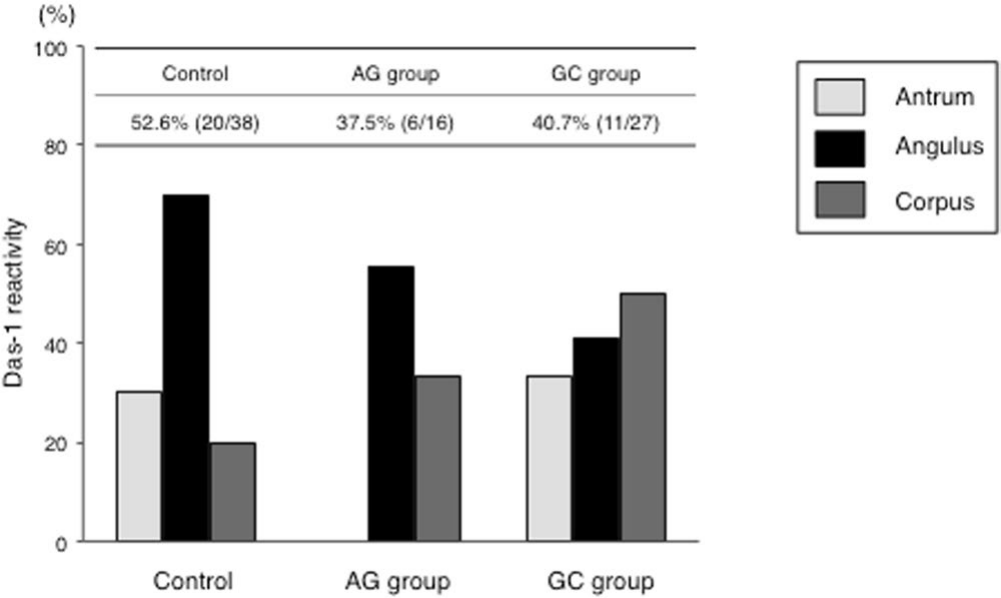
mAb Das-1 reactivity to IM in different parts of the stomach in the three groups. mAb Das-1 reactivity was not different among the three groups, but reactivity in the GC group (40.7%) was lower than that in our previous studies^32–35^.

There was no significant difference in reactivity to IM among the three groups (Fig. 2). However, mAb Das-1 reactivity against IM was highest in the angulus compared to the other portions of the stomach.

## Discussion

To the best of our knowledge, this is the first study to show the effects of aspirin on molecular alterations related to carcinogenesis in patients with chronic gastritis with and without gastric dysplasia. The patients who regularly took aspirin were followed up to 15 years. Additionally, we evaluated the molecular differences between the two precancerous conditions of AM and IM, and identified biomarkers linked with gastric dysplasia risk. We found that long-term aspirin use was associated with a significant reduction of *CDH1* methylation in AM and CIMP in IM, and that *CDH1* methylation in AM was a significant biomarker for gastric dysplasia in patients taking aspirin.

*H. pylori* infection causes aberrant DNA hypermethylation of specific genes and induces CIMP, which is an important epigenetic mechanism of tumorigenesis^37,38^. Previous studies have reported that *CDH1* methylation is strongly associated with *H. pylori* infection^20,23–25^, and it has been frequently observed in precancerous lesions^20,24,25^. Although several studies have demonstrated changes in DNA methylation after eradication of anti-*H. pylori* ^23–27^, no study has examined aberrant DNA methylation status in patients with chronic gastritis taking aspirin, especially taking into consideration the presence or absence of IM in atrophic gastritis. This study clearly demonstrated that the effects of aspirin in preventing of GC might be due to the reversal of aberrant methylation, as is the case with *H. pylori* eradication. Tahara *et al*.^39^ showed that chronic aspirin use was associated with a lower risk of *CDH1* methylation in *H. pylori*-positive subjects, similar to the findings of our study. However, in that study, the duration of aspirin use was relatively short, and methylation status was only evaluated in biopsy specimens taken from the antrum. Moreover, the authors performed analysis of methylation status using methylation-specific PCR, which is prone to false-positive results and is qualitative. In contrast, the methylation-sensitive high resolution melting (MS-HRM) analysis used in our study is applicable for the sensitive and quantitative assessment of methylation levels in an unmethylated background^33,40^.

Interestingly, frequent aberrant hypermethylation was significantly increased in the GC group compared to the AG group, although most CpG island methylation was lower in the AG group than in the controls. Once methylation has occurred in a cell, it is difficult to conceive that demethylation can occur again in the same region (temporary methylation). Residual aberrant methylation, even after aspirin use, is thought to reflect methylation in gastric gland stem cells (permanent methylation)^41^; thus, individuals with residual methylation may be at risk of GC. Although the disappearance of *CDH1* methylation is important for preventing the development of GC^20,23–26^, *CDH1* methylation that persists after long-term aspirin use may be a surrogate marker of GC.

In this study, the number of genes that changed from methylated to unmethylated after aspirin use was higher in AM than in IM, indicating that aspirin was less effective in reversing the methylation that occurred in IM compared to AM. One reason for this difference may be the methodology of DNA extraction between AM and IM; DNA from AM was extracted from whole biopsy tissues with inflammatory cells, whereas DNA from IM was only extracted from IM glands using laser microdissection. Thus, the decrease in methylation levels observed in AM specimens is probably due to cell turnover caused by the anti-inflammatory effects of aspirin (temporary methylation)^41^. Although both AM and IM are considered to be precancerous conditions and important risk factors for GC^42–45^, it is not known as which lesion has advanced potential for GC^44,46^. Our study showed that all molecular events except *CDH1* methylation were more frequently observed in IM than in AM, indicating that IM might have a more aggressive state than AM with regard to molecular alterations. Taken together, these results are in agreement with the concept of “point of no return”^47^, in which the benefits of aspirin diminish after the development of IM accompanied with molecular changes.

The accumulation of aberrant DNA methylation in non-cancerous tissues was recognized as “epigenetic field for cancerization”, especially in inflammation-associated cancers such as GC^38,41,48,49^. In this study, methylation was analyzed in biopsy specimens taken from three different parts of the stomach, as in the study by Perri *et al*.^25^. Most of the molecular alterations other than cyclin-dependent kinase inhibitor 2A (*CDKN2A*) and *MLH1* gene methylation were identified particularly in IM in the AG and GC groups for all portions of the stomach, which strongly supports the concept of “epigenetic field for cancerization”. Furthermore, the frequency of *CDH1* methylation in AM, a biomarker of GC in our study, at the greater curvature of the corpus was significantly higher in the GC group than in the AG group, suggesting that *CDH1* methylation in biopsies from this region is a potential biomarker of GC.

In this study, no *MLH1* methylation was seen in the background mucosa with MSI. We did not analyze *MSH2* and other DNA mismatch repair (MMR) genes; therefore, alterations in MMR genes except *MLH1* methylation may be associated with the mechanism underlying MSI in the precancerous lesions. We demonstrated that long-term aspirin use decreased CIMP in IM. CIMP is commonly considered to be a phenotype of GC^30,37,38^, and *H. pylori* infection causes aberrant DNA hypermethylation of specific genes and induces CIMP, an important epigenetic mechanism of gastric tumorigenesis^38^. However, there is little evidence of CIMP in precancerous lesions, although a study showed that CIMP status in GCs did not correlate with methylation levels in the background gastric mucosa^50^. Therefore, we investigated CIMP, which exhibits widespread CpG island methylation, in precancerous lesions.

We previously reported highly significant reactivity of mAb Das-1 against IM in GC patients compared to IM from non-cancer patients^32–35^. In addition, *H. pylori* eradication did not reduce the histologic IM score, but rather, changed the cellular phenotype of IM as identified by this mAb. In this study, immunoreactivity against mAb Das-1 in the GC group was relatively lower (40.6%) than that shown in our previous study (58–82%)^32–35^. This phenomenon may indicate that aspirin causes changes in colonic phenotype in GC patients. However, the results showed that GC developed even in the presence of reduced Das-1 reactivity, indicating that other neoplastic biomarkers including epigenetic alterations in addition to changes in cellular phenotype seem likely to underlie GC. In our previous studies^32,33,35^, we evaluated mAb Das-1 reactivity in biopsy specimens obtained from the greater curvature of the antrum and corpus. In this study, mAb Das-1 reactivity in the control group was similar to that in our previous reports when evaluating samples taken from those two regions; however, reactivity at the angulus was higher than that in the other two parts.

There were some limitations in this study. First, this was a study from a single institution with a small number of patients. Second, this was a cross-sectional study, which inevitably includes various types of biases. However, a randomized prevention trial to determine the preventive effects of aspirin in GC may be impossible due to the cost, time, and risk of adverse events such as gastrointestinal bleeding.

In conclusion, we found that long-term use of aspirin for more than 3 years decreases *CDH1* methylation in AM and CIMP in IM, and that methylation of the *CDH1* gene in AM is a surrogate marker of GC in patients regularly taking aspirin. In addition, IM might be more aggressive than AM with regard to molecular alterations. Our results indicate that changes in molecular events may explain the chemopreventive effects of aspirin in decreasing the incidence of GC. A prospective study in patients who are at “high risk” for GC is needed.

## Patients, Materials, and Methods

### Patients

We conducted a cross-sectional study between March 2011 and December 2016 enrolling consecutive patients with dysplasias comprising gastric adenomas or other GCs who received endoscopic resection (ER) at Hyogo College of Medicine Hospital (Hyogo, Japan). During this period, 522 consecutive patients with a total of 615 dysplasias comprising gastric adenomas (n = 50) and other GCs (n = 565) were treated with ER. Among them, 22 patients (4.2%) who had taken aspirin or NSAIDs for more than 3 years developed primary gastric dysplasia. However, because 4 of these 22 patients refused to enroll in this study, the finally study population comprised 18 patients in the GC group. The histology of these 18 primary gastric dysplasias was adenoma in 2, well differentiated-type in 13, moderately differentiated-type in 2, and poorly differentiated-type adenocarcinoma in 1. We analyzed these 18 patients who had developed primary gastric dysplasia despite taking low-dose aspirin (100 mg/day) or NSAIDs for more than 3 years (GC group); patients with histologically atrophic gastritis who regularly took low-dose aspirin or NSAIDs for more than 3 years (AG group); and patients with atrophic gastritis who did not take aspirin between October 2015 and December 2016 (control group). We used the criteria of the Japanese Research Society for Gastric Cancer^51^ as the histological criteria in this study. Patients with a history of esophagectomy or gastrectomy were excluded.

### Consent and institutional review board

Written informed consent was obtained from all of the patients prior to this study. The Ethics Committee of Hyogo College of Medicine approved this study (Nos Rin-Hi 136 and 300). This trial is registered with the UMIN Clinical Trials Registry (No. UMIN000021857). The study was performed in accordance with the Declaration of Helsinki.

### *H. pylori* status and DNA extraction

During each patient’s endoscopy, three biopsy specimens were taken from the greater curvatures of the antrum and corpus and the lesser curvatures of the angulus (one from each site). Each biopsy specimen was used for histologic examination by hematoxylin and eosin staining, Giemsa staining, mAb Das-1 staining, and DNA extraction. *H. pylori* status was analyzed in each patient with the following methods: urea breath test, Giemsa staining, and the E-plate anti-*H pylori* IgG antibody test (Eiken Kagaku, Tokyo, Japan). A patient was regarded as *H. pylori-*positive if the result of at least one of the three aforementioned methods was positive. From the paraffin-embedded biopsy specimens, two or three 7 µm thick tissue sections were cut for DNA extraction. DNA was extracted from goblet IM (incomplete type) using the QIAamp DNA Micro Kit (Qiagen, Hilden, Germany). Goblet IM was isolated using the PALM MicroBeam laser microdissection system (Microlaser Technologies, Munich, Germany) to avoid DNA contamination of inflammatory or stromal cell nuclei^32,33^ (Supplementary Fig. S1). In contrast, DNA from AM without IM was extracted from whole biopsy tissues, and as such, these samples might have contained inflammatory cells. One sample obtained from the angulus in the GC group could not be analyzed for molecular alterations due to the small amount of DNA extracted from the biopsy specimen. Finally, a total of 221 biopsy samples from 74 patients were analyzed in this study. In this cross-sectional study, we investigated molecular events including MSI, methylation of CpG islands of various genes, CIMP, and mAb Das-1 reactivity.

### Analysis of MSI by high-resolution fluorescent microsatellite analysis

As previously reported^32,33,47^, we examined the following five microsatellite loci on chromosomes for MSI based on the revised Bethesda panel^52^: BAT26, BAT25, D2S123, D5S346, and D17S250. The PCR products were evaluated for MSI by capillary electrophoresis using an ABI 3130xl Genetic Analyzer (Applied Biosystems, Foster City, CA, USA) and automatic sizing of the alleles using a GeneMapper^®^ Ver. 4.0 (Applied Biosystems). The MSI status was judged according to previous reports^40,53,54^ (Supplementary Fig. S2). In cases that were indistinguishable between MSI and loss of heterozygosity^54^, the allelic imbalance (AI) ratio was calculated. MSI was determined to be positive when the AI ratio (normal allele 1:normal allele 2/tumor allele 1:tumor allele 2) was <0.67 or >1.35, as previously reported^40,53^ (Supplementary Fig. S2). Lesions were defined as MSI in two or more of the five investigated markers^40^.

### Sodium bisulfite modification of DNA and CIMP markers

Similar to previous reports^33,40^, purified DNA samples were chemically modified by sodium bisulfite with an EpiTect^®^ Fast Bisulfite Kit (Qiagen). The bisulfite-modified DNA was amplified using primer pairs that specifically amplify the methylated or unmethylated sequences of several genes/loci related to carcinogenesis including *CDH1*, *CDKN2A* (*p16*), *MLH1*, MINT1, MINT31, *O6-methylguanine-DNA methyltransferase* (*MGMT*), and *runt-related transcription factor 3* (*RUNX3*). Although there are two major CIMP panels, the classic panel includes MINT1, MINT2, MINT31, *CDKN2A*, and *MLH1* ^55^; and the novel marker panel includes *CACNA1G*, *IGF2*, *NEUROG1*, *RUNX3*, and *SOCS1* ^56^. There is no gold standard with respect to gene panels and the number of marker thresholds used to define CIMP^57^. CIMP status generally implies methylation in at least two MINTs or target genes such as *p14*, *p16*, or *MLH1* when a small panel of markers is needed^58,59^. Therefore, we analyzed CIMP with use of the seven panels based on our previous report^40^.

#### MS-HRM

We performed MS-HRM analysis as previously described^33,40^. Briefly, PCR amplification and MS-HRM analysis were performed using a LightCycler^®^ 480 System II (Roche, Mannheim, Germany). The primer sequences of all genes for the methylated and unmethylated forms and PCR and MS-HRM conditions are summarized in Supplementary Tables S1 and S2. Percentages of methylation (0%, 10%, 50%, and 100%) were used to make the standard curve (Supplementary Fig. S3). In this study, only samples with >10% methylation were considered methylated. CIMP was defined as ≥3/7 methylated markers using the seven-marker CIMP panel.

### Immunoperoxidase assays with mAb Das-1

Serial sections were stained with the mAb Das-1 (a highly specific IgM mAb against the colonic phenotype), which does not react with normal gastric mucosa and atrophic mucosa other than IM, using sensitive immunoperoxidase assays as previously described^32–35^. Greater than 10% of IM glands stained with mAb Das-1 was considered positive expression (Supplementary Fig. S4).

### Statistical analysis

Continuous and categorical data are reported as means and standard deviations (SDs) and frequencies with proportions, respectively. The data were assessed by the Mann-Whitney *U-*test for comparisons between two independent groups, by the Kruskal-Wallis test for comparisons among the three independent groups, and by the chi-squared test or Fisher’s exact test for comparisons of proportions. We developed logistic regression models to assess the effect of molecular markers for gastric dysplasia. Molecular markers with a *p* value < 0.1 in univariate logistic regression model were included in the multivariate logistic regression model, followed by backward selection. The effects of molecular markers were expressed by ORs and 95% CIs. P values less than 0.05 were considered statistically significant. Statistical analyses were performed with StatView version 5.0 (SAS Institute Inc., Cary, NC, USA).

## Electronic supplementary material

Supplementary Information
