# Supplementary material for: Effects of long-term aspirin use on molecular alterations in precancerous gastric mucosa in patients with and without gastric cancer
Source: Sci Rep. 2017 Oct 17;7:13384. doi: 10.1038/s41598-017-13842-x (PMC5645329; doi:10.1038/s41598-017-13842-x)
Supplement: Supplementary file 1 — Supplementary Information [file 41598_2017_13842_MOESM1_ESM.doc]

**Effects of long-term aspirin use on molecular alterations in precancerous gastric mucosa in patients with and without gastric cancer**

Yuki Michigami^1^, Jiro Watari^1^, Chiyomi Ito^1^, Ken Hara^1^, Takahisa Yamasaki^1^, Takashi Kondo^1^, Tomoaki Kono^1^, Katsuyuki Tozawa^1^, Toshihiko Tomita^1^, Tadayuki Oshima^1^, Hirokazu Fukui^1^, Takeshi Morimoto^2^, Kiron M. Das^3^ & Hiroto Miwa^1^

^1^ Division of Gastroenterology, Department of Internal Medicine, Hyogo College of Medicine, Nishinomiya, Japan; ^2^ Department of Clinical Epidemiology, Hyogo College of Medicine, Nishinomiya, Japan; ^3^ Division of Gastroenterology and Hepatology, Departments of Medicine and Pathology, Robert Wood Johnson Medical School, Rutgers, Cancer Institute of New Jersey, New Brunswick, United States

**Supplementary Figure Legends**

**Supplementary Fig. S1.** A: The glands of incomplete-type intestinal metaplasia (IM) were isolated by laser microdissection. B: The same section after the removal of metaplastic glands.

**Supplementary Fig. S2.** Examples of MSI detected in AM by high-resolution fluorescent microsatellite analysis. (A) MSI on D2S123 is identified by the appearance of multiple additional peaks (arrows). (B) MSI on BAT25 is seen as an unequivocal extra peak shift (asterisk) compared with control. (C) T1 and N1 represent the highest respective peak areas of the shorter allele in AM, and control samples and T2 and N2 represent the highest respective peak areas of the longer allele. MSI on D5S346 (longer allele, T2) of AM DNA is seen. The allelic imbalance ratio is 2.07. (D) MSI on D2S123 is seen by the disappearance of longer allele (arrow).

**Supplementary Fig. S3.** Representative results of MS-HRM analysis for methylation. Results are shown for the *CDH1* gene with positive (fully methylated) and negative controls (fully unmethylated), and a biopsy sample. The melting peaks were calculated from melting curves of HRM. Each sample was directly compared with its control to identify the sample’s methylation status, and the differences in fluorescence between samples were normalized by the analysis algorithms. Methylated and partially methylated DNA (≥ 10%) were considered to be positive for methylation, and unmethylated DNA was treated as negative. The sample shows a moderate level of methylation (≥ 10% to <50%).

**Supplementary Fig. S4.** Representative section of immunoperoxidase staining with mAb Das-1. Das-1 reactivity in the IM was demonstrated in the GC group (X200).


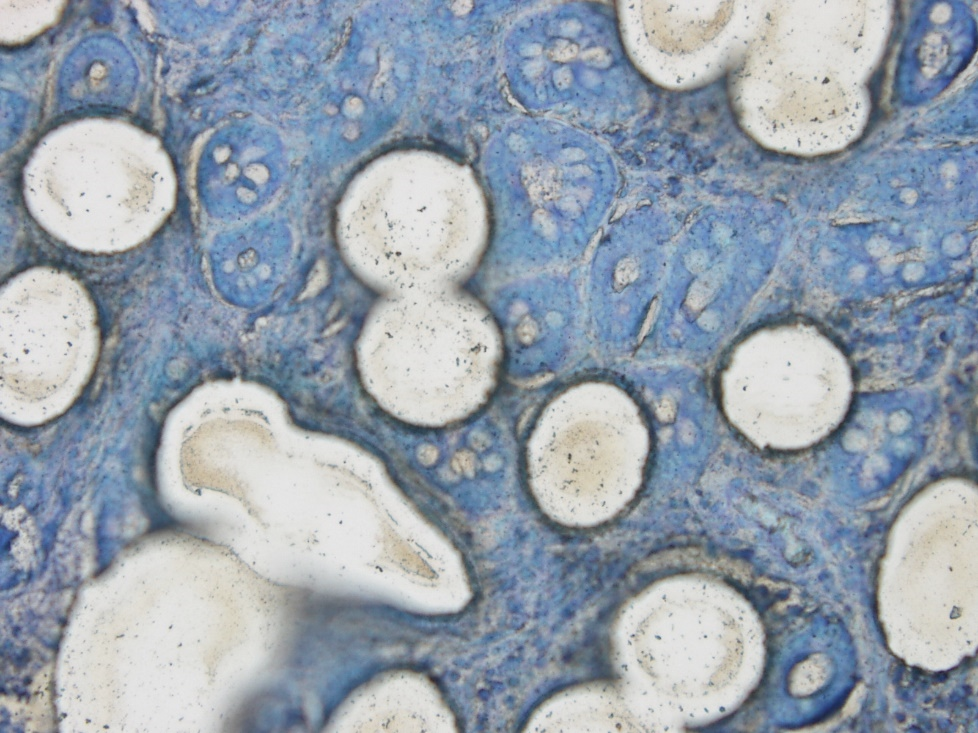


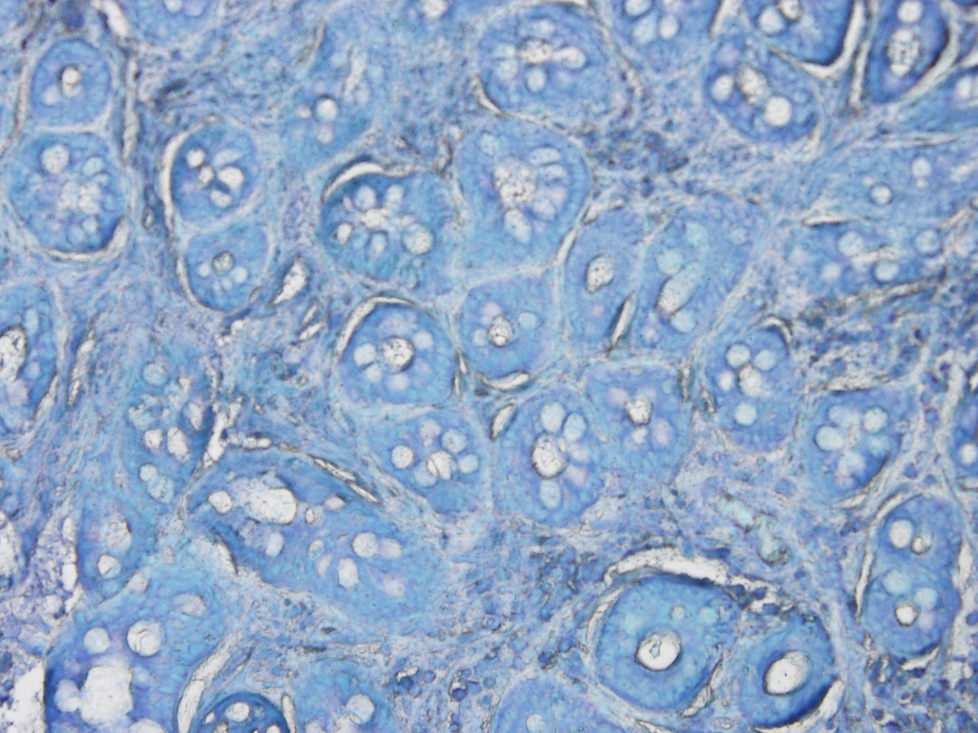


IM

IM

IM

IM

IM

IM

IM

**A**

**B**

**Supplementary Fig. S1**


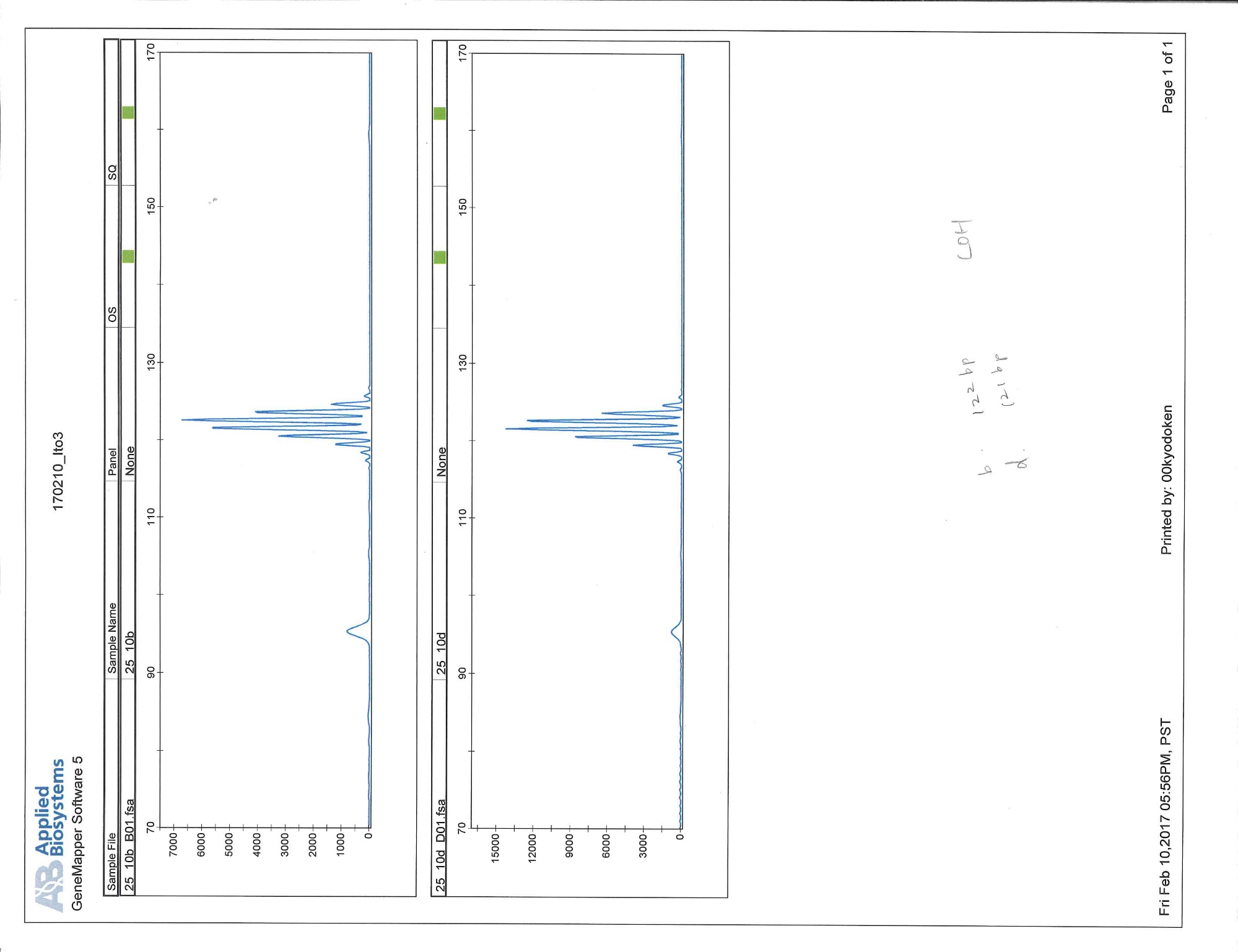

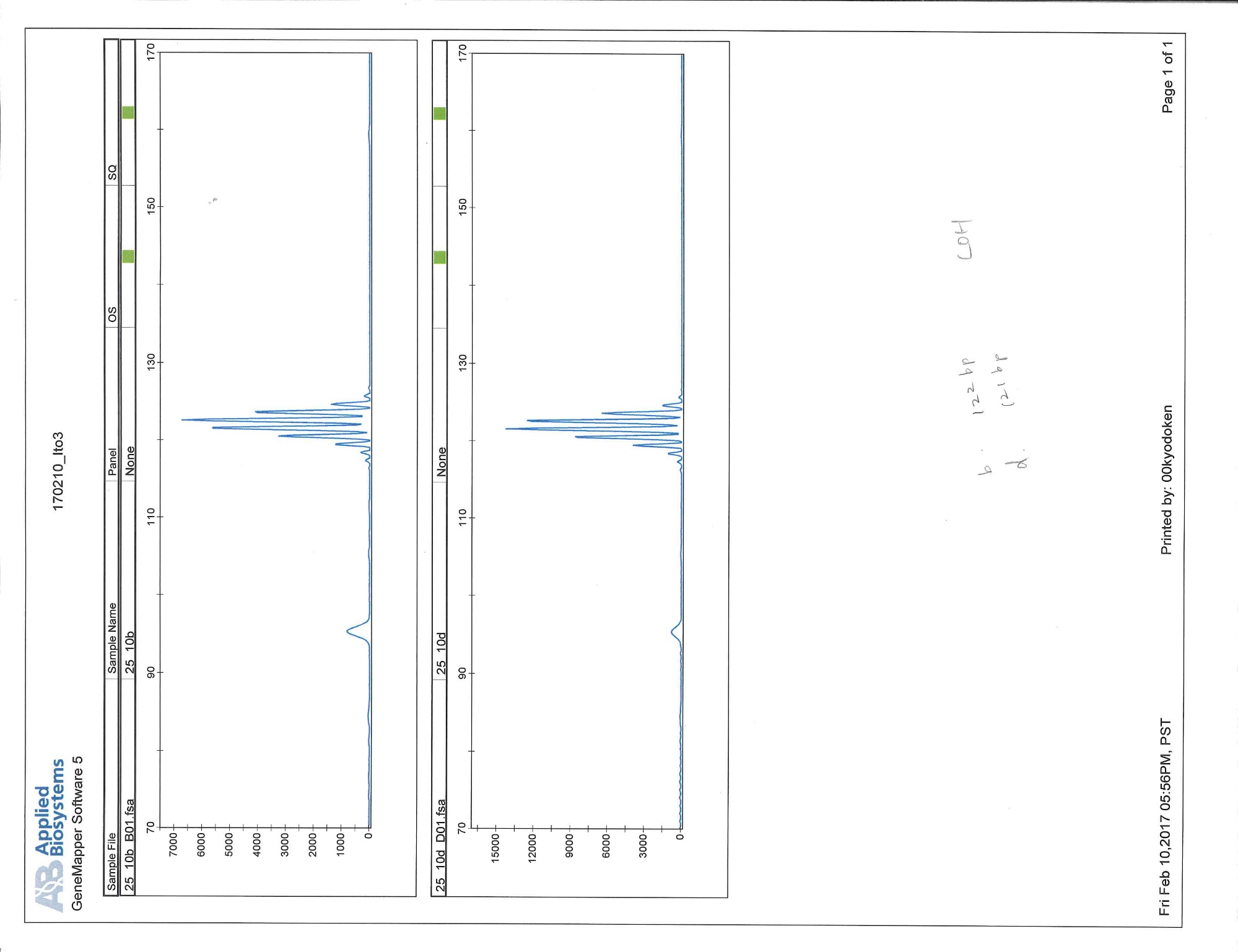

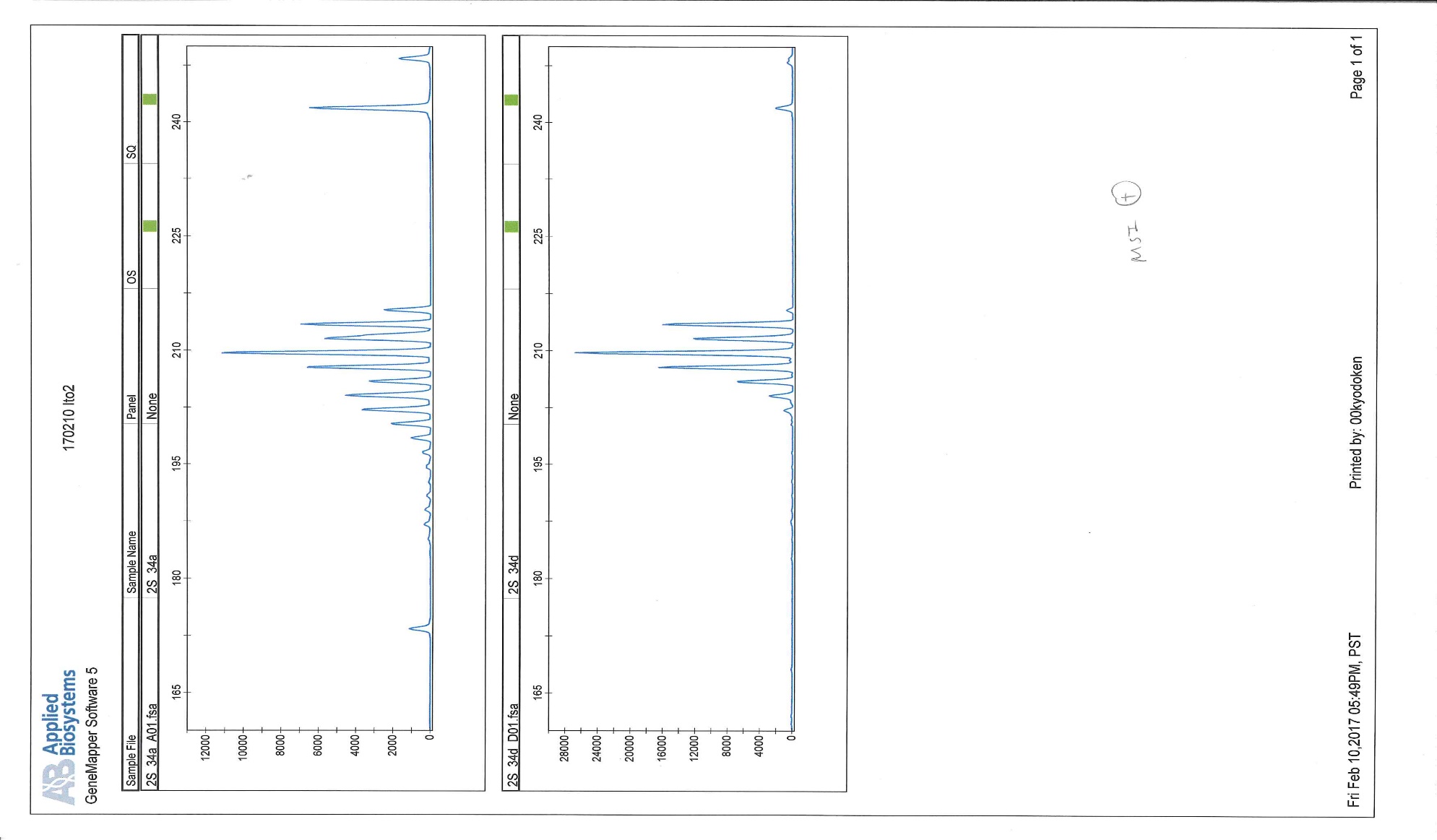


**D**


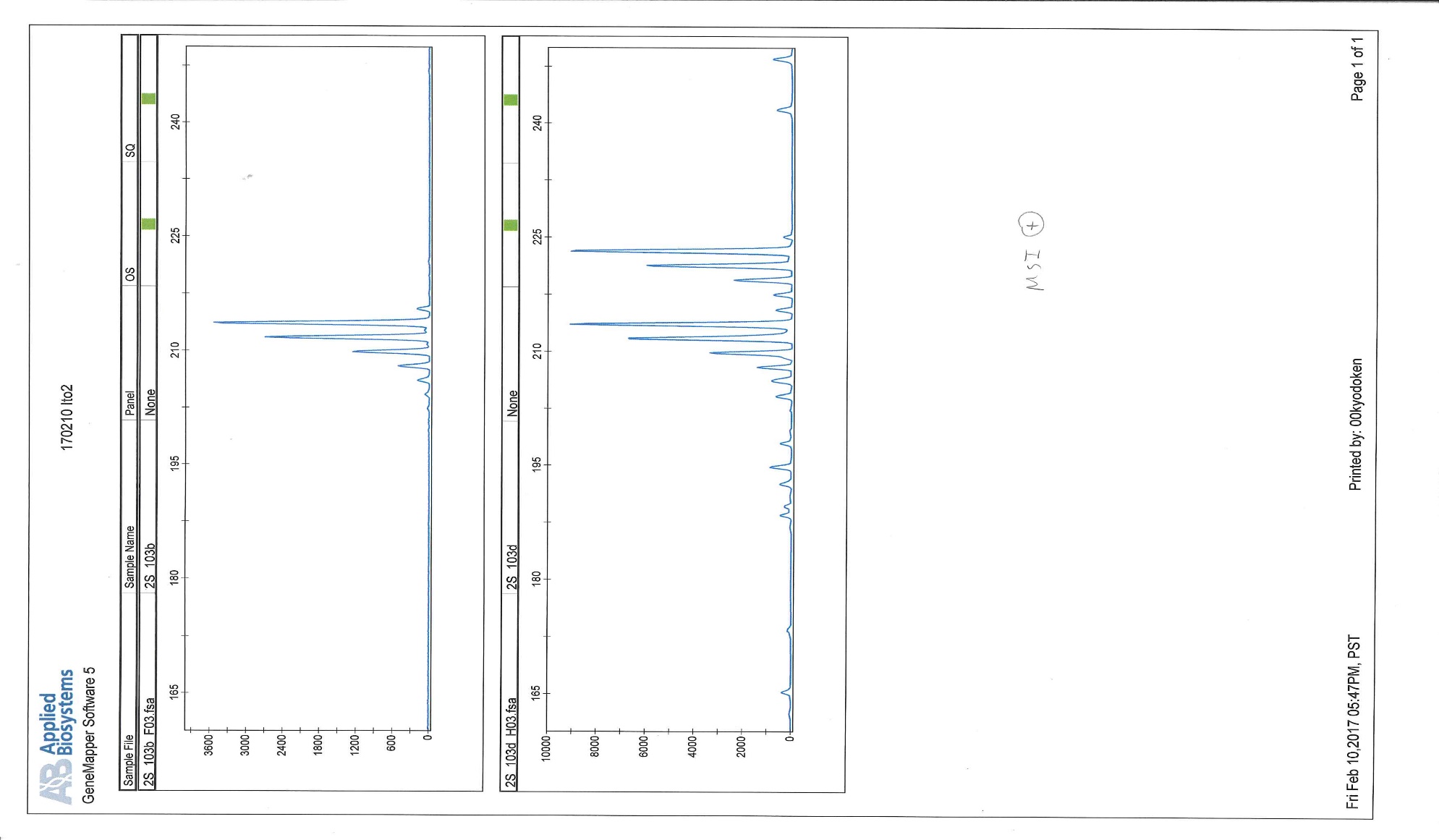

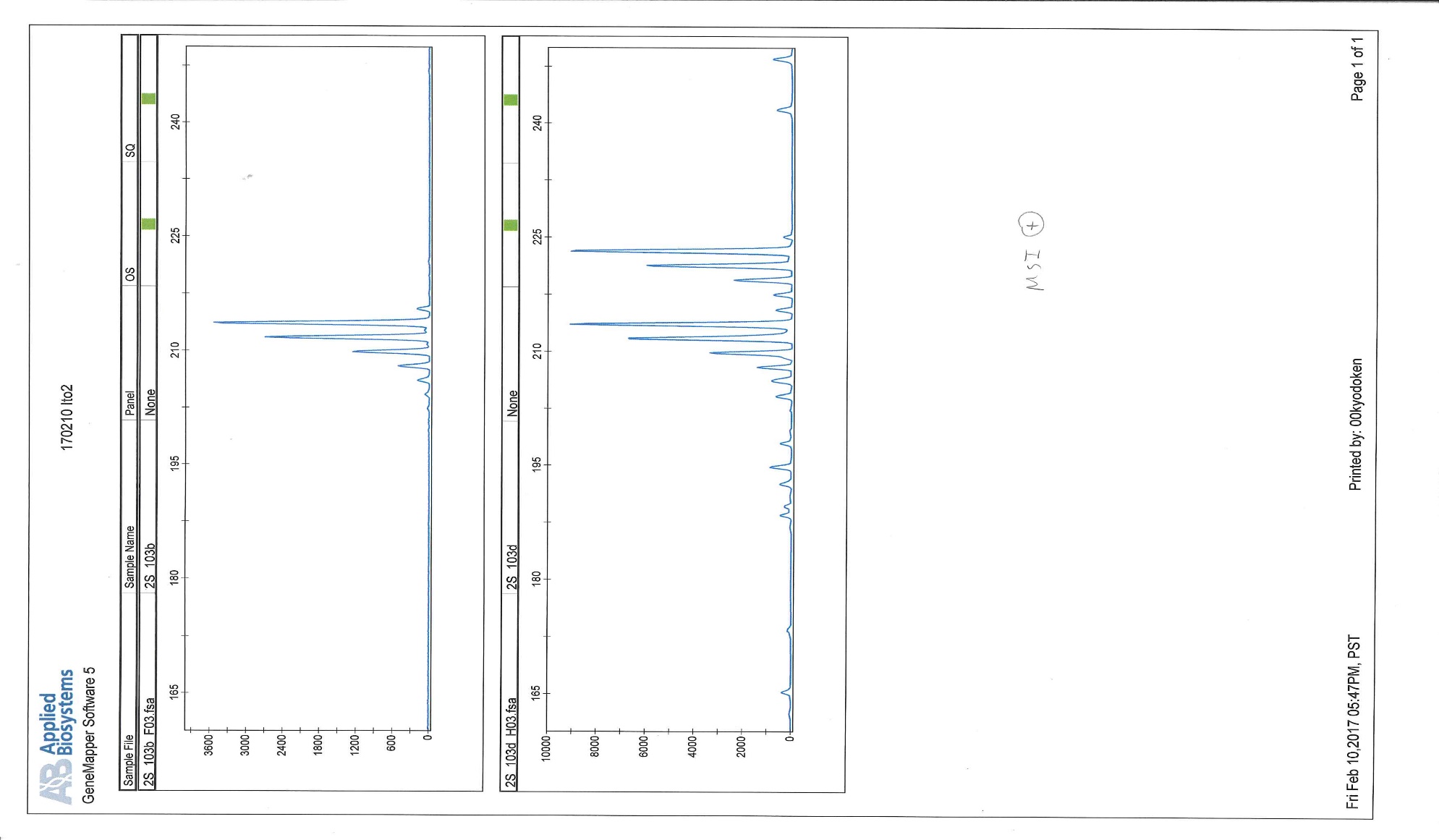


(base pairs)

Control

Sample

(base pairs)

Control

Sample

**B**

**A**

(base pairs)

**C**

**Supplementary Fig. S2**


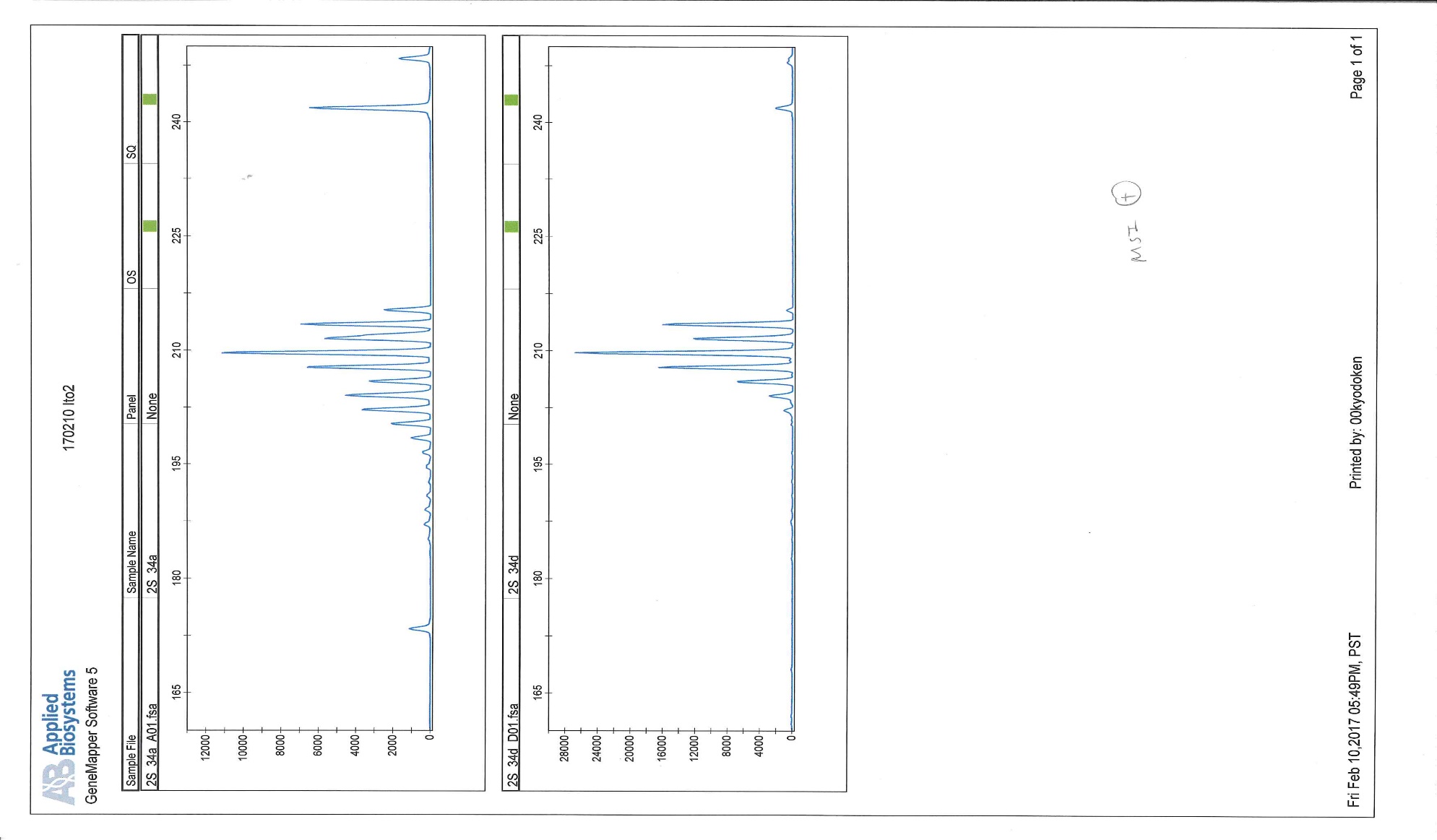

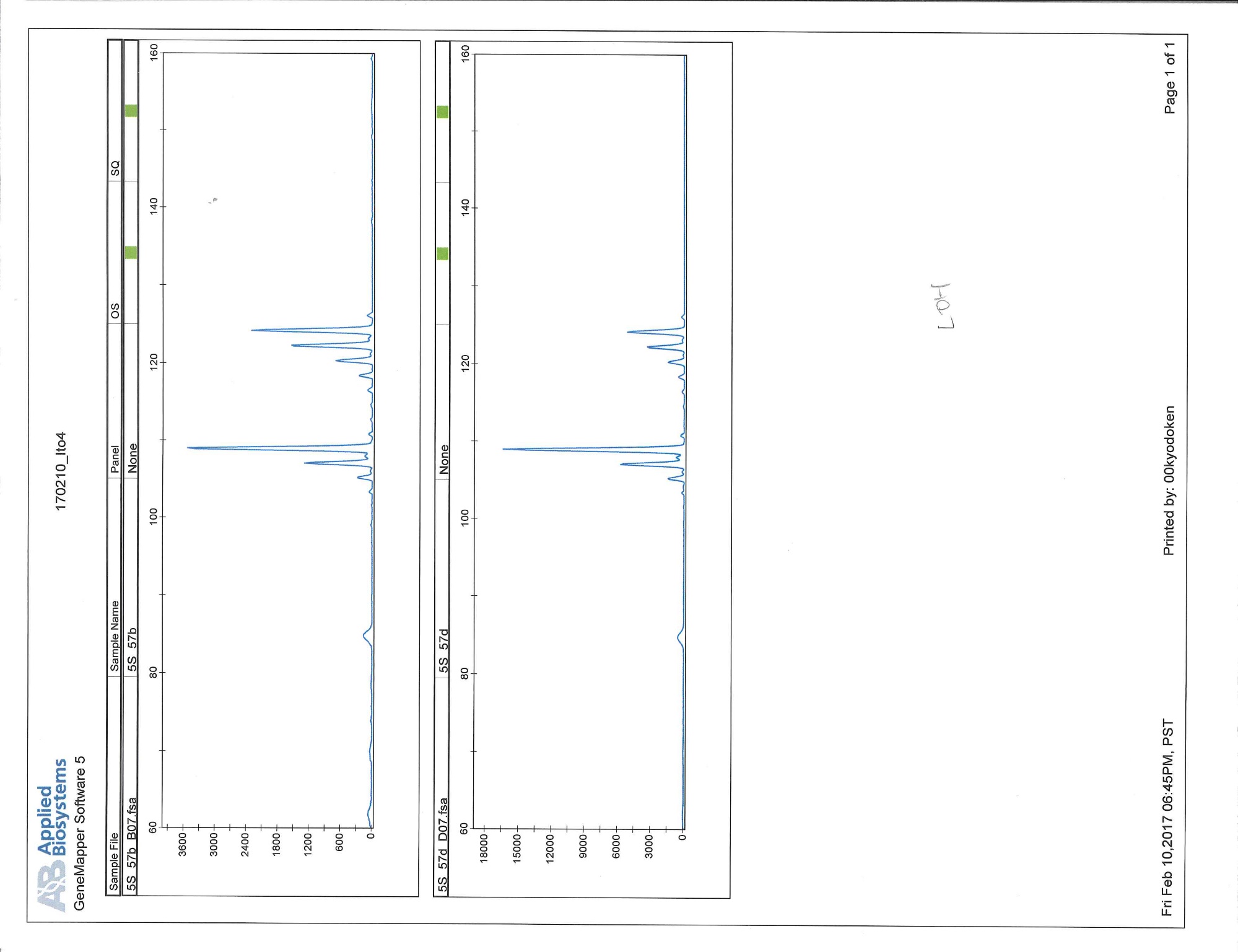

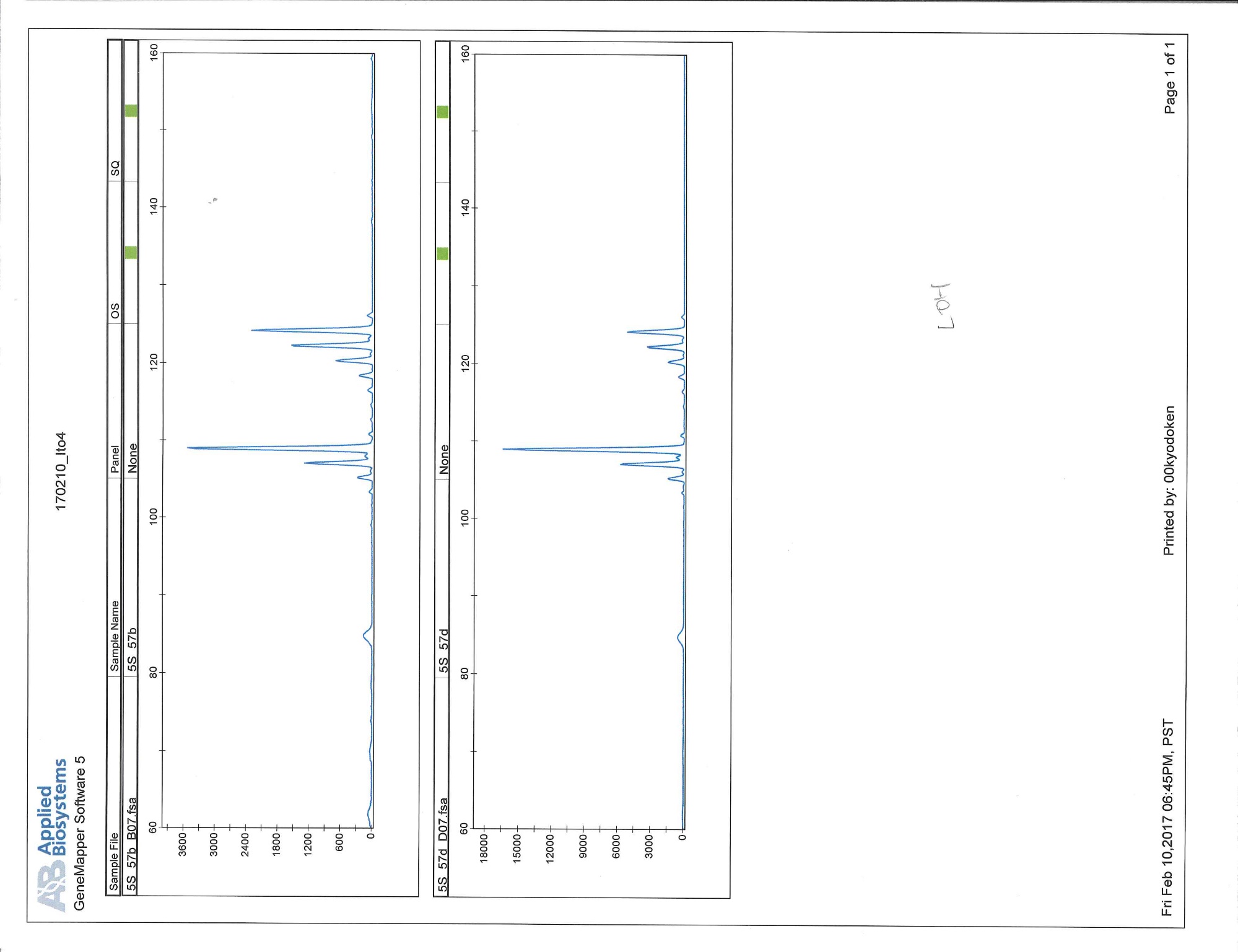


Control

Sample

(base pairs)

Control

Sample

N1

N2

T2

T1

*


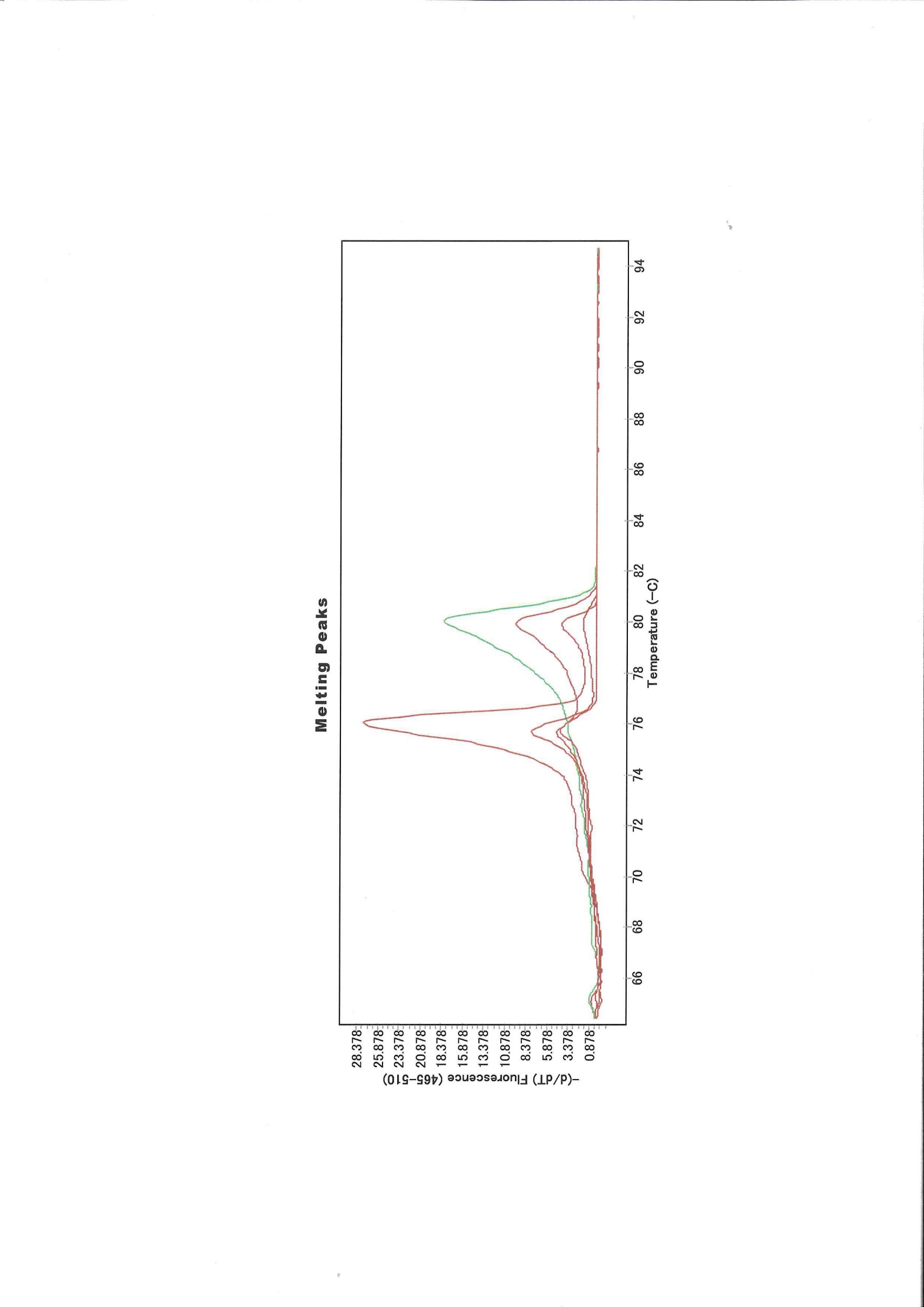


**Supplementary Fig. S3**

100% Methylated

10% Methylated

50% Methylated

0% Methylated

Sample


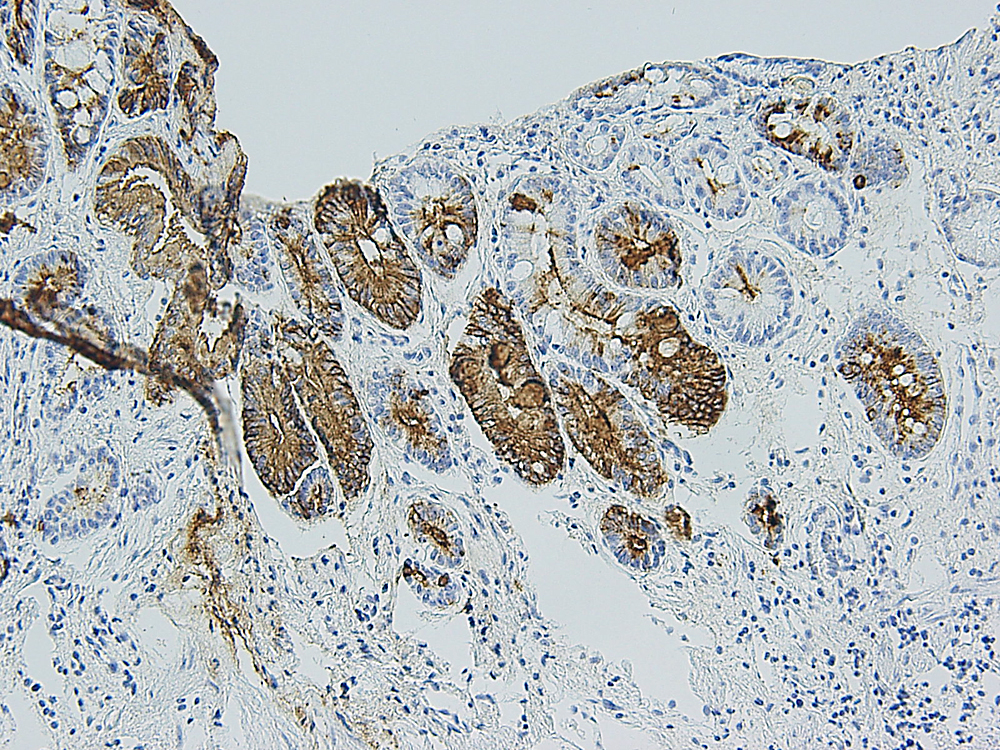


**Supplementary Fig. S4**

Supplementary Table 1. Primer sequences for the MS-HRM assays

| Gene | Primer sequences (5’ – 3’) |
| --- | --- |
| *CDH1* | F - GGAATTGTAAAGTATTTGTGAGTTTG |
|  | R - AAAATACCTTCAACCAATCACCTC |
| *CDKN2A* | F- CGGAGGAAGAAAGAGGAGGGGT |
|  | R- CGCTACCTACTCTCCCCCTCT |
| *MLH1* | F - TTGGTATTTAAGTTGTTTAATTAATAGTTG |
|  | R - AAAATACCTTCAACCAATCACCTC |
| *MGMT* | F- GTTTCGGGTTTCGTATTTATTTTGAAGG |
|  | R- GACAACCCCAACTTCCTCTACTC |
| MINT1 | F- GGGGTTGAGGTTTTTTGTTAG |
|  | R- AATCCCTCTCCCCTCTAAACTT |
| MINT31 | F- GGGTGATGGTTTTAGTAAAGTGAG |
|  | R- AAAAACACTTCCCCAACATCTAC |
| *RUNX3* | F- GTTTCGGGTTTCGTATTTATTTTGAAGG |
|  | R- GACAACCCCAACTTCCTCTACTC |

**Supplementary Table 2.** PCR and MS-HRM conditions in each gene

| Pre-incubation | | |  |  | Amplification | | | | | |  |  | Cooling | | |
| --- | --- | --- | --- | --- | --- | --- | --- | --- | --- | --- | --- | --- | --- | --- | --- |
| Denaturation (°C) | Hold  (min) | Ramp rate  (°C/s) |  |  | Target  (°C) | Hold  (sec) | Ramp rate  (°C/s) | Sec target^*^  (°C) | Step size^*^  (°C) | Step delay^*^  (°C) | Cycles |  | Target  (°C) | Hold  (sec) | Ramp rate  (°C/s) |
| 95 | 10 ^a,b,c,d,e,f^  15 ^g^ | 4.8 |  | Denaturation | 95 | 10 ^c,d,e,f^  15 ^g^  30 ^a,b^ | 4.8 |  |  |  | 45 ^a,b,d,e^  50 ^c,f,g^ |  | 40 | 30 | 2.5 |
|  |  |  |  | Annealing | 51 ^d^, 55 ^g^  62 ^e^, 63 ^b^  64 ^c^,65 ^a,f^ | 10 ^d,e^  15 ^g^  30 ^a,b,c,f^ | 2.5 | 50 ^e^, 52 ^c^  53 ^f^, 57 ^b^  60 ^a^ | 0.5 ^a,b,c,e,f^ | 1 ^a,b,c,e,f^ |  |  |  |  |
|  |  |  |  | Extension | 72 | 10 ^d,e^  15 ^b^  20 ^g^  30 ^a,c,f^ | 4.8 |  |  |  |  |  |  |  |
|  |  |  |  |  |  |  |  |  |  |  |  |  |  |  |  |
|  |  |  |  |  | High-resolution melting | | | | | |  |  |  |  |  |
|  |  |  |  | Denaturation | 95 ^a,b,c,d,e,f^  97 ^g^ | 10 ^b^  60 ^a,c,d,e,f,g^ | 4.8 |  |  |  |  |  |  |  |  |
|  |  |  |  | Annealing | 40 ^a,c,d,e,f,g^  50 ^b^ | 60 | 2.5 |  |  |  |  |  |  |  |  |
|  |  |  |  | Melting interval | 65 | 1 ^a,c,d,e,f,g^  15 ^b^ | 4.8 |  |  |  |  |  |  |  |  |
|  |  |  |  | Continuous | 95 | - | 0.02 | Acquisition 25 (/°C) | | |  |  |  |  |  |

^*^ Touchdown method, ^a^ *CDH1*, ^b^ *CDKN2A*, ^c^ *MLH1,* ^d^ *MGMT*, ^e^ MINT1*,* ^f^ MINT31*,* ^g^ *RUNX3*
